# The Effect of the Teach-Back Method on Knowledge, Performance, Readmission, and Quality of Life in Heart Failure Patients

**DOI:** 10.1155/2020/8897881

**Published:** 2020-11-23

**Authors:** Ali Rahmani, Amir Vahedian-Azimi, Masoud Sirati-Nir, Reza Norouzadeh, Hamid Rozdar, Amirhossein Sahebkar

**Affiliations:** ^1^Trauma Research Center, Nursing Faculty, Baqiyatallah University of Medical Sciences, Tehran, Iran; ^2^Behavioral Sciences Research Center, Life Style Institute, Nursing Faculty, Baqiyatallah University of Medical Sciences, Tehran, Iran; ^3^Nursing and Midwifery faculty, Shahed University, Tehran, Iran; ^4^Biotechnology Research Center, Pharmaceutical Technology Institute, Mashhad University of Medical Sciences, Mashhad, Iran; ^5^Neurogenic Inflammation Research Center, Mashhad University of Medical Sciences, Mashhad, Iran; ^6^Halal Research Center of IRI, FDA, Tehran, Iran

## Abstract

**Background:**

Among chronic diseases, heart failure has always been a serious challenge imposing high costs on health systems and societies. Therefore, nurses should adopt new educational strategies to improve self-care behaviors and reduce the readmissions in heart failure patients. This study aimed to determine the effect of the teach-back method on knowledge, performance, readmission, and quality of life in these patients.

**Methods:**

This clinical trial was conducted in patients with heart failure (*n* = 70) hospitalized in the internal wards of the Baqiyatallah al-Azam Medical Center in Tehran (2019). Routine discharge educations were provided in control patients. Self-care topics were taught to the intervention groups by the teach-back method. A cardiac self-care questionnaire was used to assess the knowledge and practice of patients immediately after intervention and three months after patient discharge. Also, SF-36 was presented to each patient. Readmission(s) and quality of life were followed up by telephone interviews three months after patient discharge. Repeated measures analysis of variance and related post-hoc tests were performed for within-group comparisons before, immediately after, and 3 months after teach-back education. Wilks' lambda multivariate tests were conducted for simultaneous comparison of quality of life subscales between intervention and control groups. Also, logistic regressions were after controlling for baseline measures and confounders.

**Results:**

Findings showed significant improvement in the patients' knowledge and performance immediately after teach-back education, though this effect was slow in the long term after discharge. Also, the frequency of readmissions decreased and the quality of life (except physical function) increased in the patients through teach-back education. By controlling for the pretest effect, the posttest scores for the relevant components of the quality of life suggested improvement in both intervention and control patients. This improvement in the quality of life was confirmed by controlling for baseline measurements using binary logistic regression analysis.

**Conclusion:**

Teach-back education improved patients' knowledge and performance, readmission frequency, and quality of life.

## 1. Introduction

Chronic disease is a physical or mental condition that persists for more than a year, resulting in functional limitations and a decline in quality of life that requires ongoing monitoring or treatment [1]. Cardiovascular disease is recognized as a major cause of death in all communities [2, 3]. Among cardiovascular diseases, heart failure is one of the health problems around the world [4] with an incidence of between 1% and 2% in adults, and the global prevalence is estimated to reach 25% in 2030 [5]. The prevalence of heart failure in Iran is 8% and is higher than of the amount reported in Asia and worldwide, which is a serious challenge for Iran's health system [6]. Heart failure is an important factor for hospitalization and imposing high costs on society [7] because the cost of hospitalization of heart failure patients is very expensive and is estimated to be 50–79% more than other patients [8]. What we are seeing today in patients with heart failure is improved clinical outcomes such as reduced hospital stay, hospital mortality [9], and increased life expectancy due to new drug therapies and nonpharmacological interventions such as the implantable cardioverter defibrillator (ICD), cardiac resynchronization therapy (CRT), and surgical procedures [10]. Despite advances in heart failure treatment, existing research indicates increasing readmission rates in these patients [11]. Salehitali et al. [12] in one Iranian hospital showed that 47.3% of total hospital beds were occupied by heart failure patients, with an average of 5.74% annual readmission [12]. It is estimated that a quarter of patients admitted for heart failure will be readmitted within one month of being discharged due to cardiovascular disease or other medical problems such as diabetes and kidney failure. Current guidelines indicate that encouraging the patient to self-care is to detect, monitor, and manage disease symptoms, which is an important way to reduce the challenge of rehospitalizing patients [13, 14], reducing symptoms, delaying disease progression, and improving patients' quality of life [15]. The findings of a systematic review by Sousa et al. [16] suggest that heart failure patients are always struggling to balance their self-care needs, but they are often unable to self-manage for their cardiac symptoms [16]. This inability to self-care can affect the quality of life of heart failure patients [17]. Heart failure patients perceive the quality of life as the ability to perform physical and social activities, stay happy, and interact with people who are affected by physical, psychological, economic, social, spiritual, and behavioral (self-care) variables [18]. Patient participation in self-care activities such as adherence to treatment regimes and timely referral to physician when symptoms increase are the influential factors affecting the quality of life in patients with heart failure. But, the reality is that most of these patients do not have the preparation and education to effective self-care [19]. Self-care education for patients is one of the most important elements of heart failure control which helps patients to have better daily life, social function, and quality of life [20]. Self-care education in these patients is associated with reduced readmission and treatment costs [21, 22], reduced mortality, increased satisfaction [23], and improved compliance and clinical outcomes [24]. In fact, systematic education provides opportunities for better self-care and self-management in patients with heart failure [25]. Nurses play an important role in educating heart failure patients and must have sufficient knowledge to prepare these patients for self-care behaviors [26]. In this regard, there are research studies on self-care education and the effects of this education on heart failure patient outcomes, including self-care based on full-course individualized health education [27], the telephone-based self-management program [28], the empowerment-based model [29], video-based training [30], and family-centered education [31]. The teach-back method is one of the new education methods to increase self-care skills. Teach-back education is a way to determine the level of understanding by asking the patient after training. This method is a useful tool to help the patient to better understand medical conditions, medication management, full participation in treatments, and adherence to protocols that enhance the quality of self-care [32]. Also, teach-back education is a strategy for evaluating the effectiveness of trained concepts and self-care ability in patients [33, 34]. In this regard, the systematic review of Ha Dinh et al. [35] shows that teach-back education improves adherence to treatment regimens, self-management, and self-care and decreases readmission of patients with chronic disease [35]. Other research studies have shown the effectiveness of teach-back education in self-management in renal transplant recipients [36], health promotion of diabetic patients [37], using respiratory inhalers in chronic pulmonary diseases [38], and postpartum quality of life [39].

In recent years, researchers have been focusing on the effects of feedback training on heart failure patients. For example, a clinical trial on 140 Vietnamese heart failure patients showed that predischarge self-care education improves their knowledge and self-care [40]. On the other hand, it has been found that teach-back education alone or in combination with film and discussion educational is associated with reduced readmission in these patients [41, 42]. Also, teach-back education enhances knowledge retention and recalling of education in heart failure patients [33]. Only one study in Iran shows the effectiveness of the teach-back method on self-care learning in heart failure patients compared to face-to-face education based on educational booklet; in any case, this study does not address the other self-care outcomes of heart failure patients [43].

One of the challenges for nurses is their uncertainty about the proper understanding of heart failure patients about the concepts of self-care. To address this concern, nurses need to have sufficient knowledge and perception of new evidence-based methods in education of patients with heart failure. This is especially important when the strategy of transferring the burden of heart failure treatment to homecare is put forward to improve the well-being and quality of life [44] and to maintain ownership of health in these patients [45]. Based on this information, the researchers were curious to study the effects of teach-back education on the perception, performance, quality of life, and readmission of heart failure patients.

## 2. Methods

### 2.1. Objective

This quasiexperimental study was performed to determine the effect of teach-back education on knowledge and practice, readmission, and quality of life in patients with heart failure.

### 2.2. Sample

The study population consisted of patients with heart failure admitted to the internal wards of Baghiyyatollah al-Azam Medical Center in Tehran. Considering that the type of medical intervention and the stage of heart failure have an impact on patients' life quality and health status, patients with advanced heart failure, defined as reduced systolic function (EF ≤ of 40%), were selected for this study [46]. Patients with advanced heart failure have severe limitations for physical activity, and less than ordinary activity causes their fatigue, palpitation, or dyspnea. Management of advanced heart failure patients is challenging because of worsening clinical symptoms, high rates of rehospitalization and mortality, and unstable condition despite receiving standard treatments [47]. Other inclusion criteria were age range of 45–85 years, at least 6 months have passed since the diagnosis of heart failure, be literate, having no cognitive problems, and native Persian-speaking patients. Participants were excluded in unwillingness, inability to cooperate, or worsening of the disease and the need for hospitalization in critical care setting. Patients were selected through convenience sampling and randomly assigned to intervention and control groups. Altman's nomogram method was used to determine the sample size. Mean and standard deviation of quality of life after intervention in the experimental and control groups were 2.89 ± 0.39 and 2.25 ± 0.21, respectively. Accordingly, effect size of 1.64 was calculated from the mean difference of the experimental and control groups after the intervention is divided by the standard deviation of the intervention group. Therefore, 32 patients were calculated in each group. Considering 10% of attrition in samples, a total of 70 heart failure patients were selected (35 patients for each group).

### 2.3. Data Collecting Instruments

Data collection instruments included demographic data sheet, SF-36 questionnaire, and cardiac self-care questionnaire. The SF-36 questionnaire was validated by Montazeri et al. [48] in Iran [48]. SF-36 is one of the most commonly used measures for health-related quality of life including eight dimensions: physical functioning, role limitation, physical problems, physical pain, general health, vitality, social function, and mental health. Scores in all subscales of this questionnaire were defined as 0–100; higher scores indicate more favorable physical or psychological well-being [49]. The cardiac self-care questionnaire is a 5-point Likert scale consisting of 16 items. The 4 items determine patient's knowledge, and 12 items are specific for performance assessment. Sullivan et al. [50] have confirmed the validity and reliability of this questionnaire [50]. In Iran, Allahverdipour et al. [51] reported a reliability of 0.87 for this questionnaire [51].

### 2.4. Intervention

The intervention was performed in a way that each patient in the control group received their routine care. In control patients, discharge education was routinely provided by a nurse or physician on medication, diet, and follow-up for referral to the heart. Patients in the control group were not educated on how to self-care with the teach-back method. The intervention groups were taught face-to-face by the teach-back method, in addition to usual care. Presented educational topics to each patient included heart failure symptoms, how to manage symptoms, salt intake, fluid intake management, exercise, medication, daily weighing, and warning signs [40]. The mean time of self-care was 30 minutes (range: 20–40 minutes). After a few minutes rest, the patient was asked to give feedback to the nurse on what he/she learned. In the process of patient feedback, if patient had misunderstandings of education, more explanations were provided by the nurse. Immediately after teach-back education and three months after discharge, the posttest was performed in intervention and control groups for knowledge and practice. Also, readmission(s) and quality of life were followed up three months after discharge by telephone interviews.

### 2.5. Ethical Considerations

The study was conducted in coordination with the hospital department and the department of internal cardiology, after obtaining the necessary legal authorization from the research deputy of Baqiyatallah-Azam University. After obtaining written informed consent, all participants were assured of the confidentiality of their information (personal and questionnaires information). Participants were informed that their participation in the research was completely voluntary and they could freely withdraw at any stage of the study. It was also attempted to maintain the patients' privacy, and the time of intervention was adjusted to the extent possible with the patient's willingness. Patients were allowed to have a family member present at their bedside at the time of education. Also, patients were informed that they would be provided with the results of the study if they are so desired.

### 2.6. Statistical Analysis

Statistical analysis was performed with SPSS software (ver.17) (SPSS Inc. IL, Chicago, USA). Normality of the numeric variables was checked using the Kolmogorov–Smirnov test. Data were presented using mean (SD), for the numeric normal and frequency (percent) for categorical variables. The between-group comparisons of baseline measures and demographic variables performed using the independent *t*-test and/or the chi-square test (with exact p value) were appropriate. For within-group comparisons, repeated measures analysis of variance (RMANOVA) was used, where before, immediately after, and postintervention measurements were taken, followed by Sidak post hoc tests. The assumption of sphericity was addressed by Mauchly's test of sphericity, and when the assumption was not satisfied, the Greenhouse–Geiser correction of *P* value was utilized. To assess the effect of intervention, the analysis of covariance (ANCOVA) was used after controlling for baseline measures and confounders in a two-step hierarchical model. Additionally, in a multivariate manner to assess the effect of intervention on the variables simultaneously, multivariate analysis of variance (MANOVA) was used considering the Wilks lambda. For binary primary outcome, the logistic regressions were utilized after controlling for baseline measures and confounders in a two-step hierarchical model. In all analyses, *P* values less than 0.05 were considered as significant.

### 2.7. Findings

In terms of gender of patients, the number of patients in both intervention and control groups was equal; in each group, 24 patients were male (68.6%) and 11 patients were females (31.4%). Overall, patients had an average age of 66.15 ± 13.3 years (Table 1).

### 2.8. Outcomes Variables

#### 2.8.1. Knowledge

**Figure 1:**
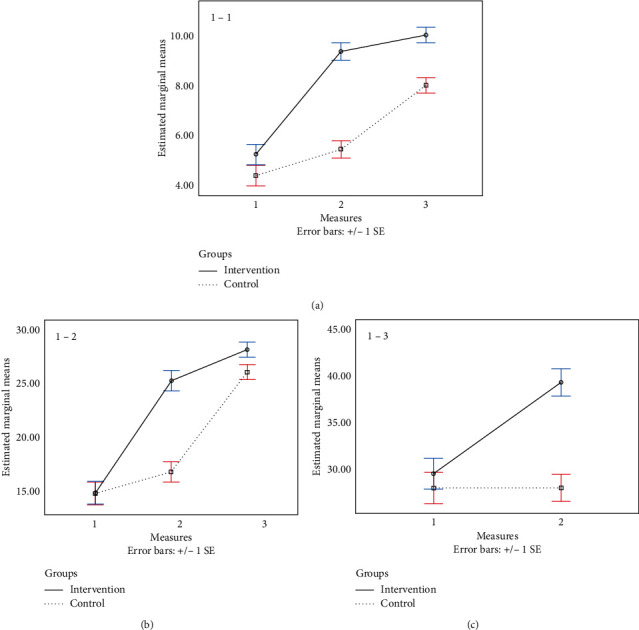
Interaction of time–group for measurements: (a) 1-1, knowledge, (b) 1-2, performance, and (c) 1–3, total SF-36.

Based on the results of two-way RMANOVA (repeated measure analysis of variance), there was a significant time-group interaction (*P* < 0.05). The results of the Sidak post hoc test indicated that, for each group, there were significant differences between all pair's measurements (*P* < 0.05). Also, the results revealed significant differences between intervention and control groups after intervention measures (*P* < 0.05). In addition, the results of ANCOVA showed that the changes in the knowledge after intervention were higher in the intervention group than that of in the control group (*P* < 0.05) as well as after adjusting for confounders (*P* < 0.05) (Table 2; Figure 1).

#### 2.8.2. Performance

Based on the results of two-way RMANOVA, there was a significant time-group interaction (*P* < 0.05). The results of the Sidak post hoc test indicated that, for each group, there were significant differences between all pair's measurements (*P* < 0.05). Also, the results revealed significant differences between intervention and control groups in after and post intervention measures (*P* < 0.05). In addition, the results of ANCOVA showed that the changes in the performance in after and post intervention were higher in the intervention group than that of in the control group (*P* < 0.05) as well as after adjusting for confounders (*P* < 0.05) (Table 2, Figure 1).

#### 2.8.3. Physical Function

**Figure 2:**
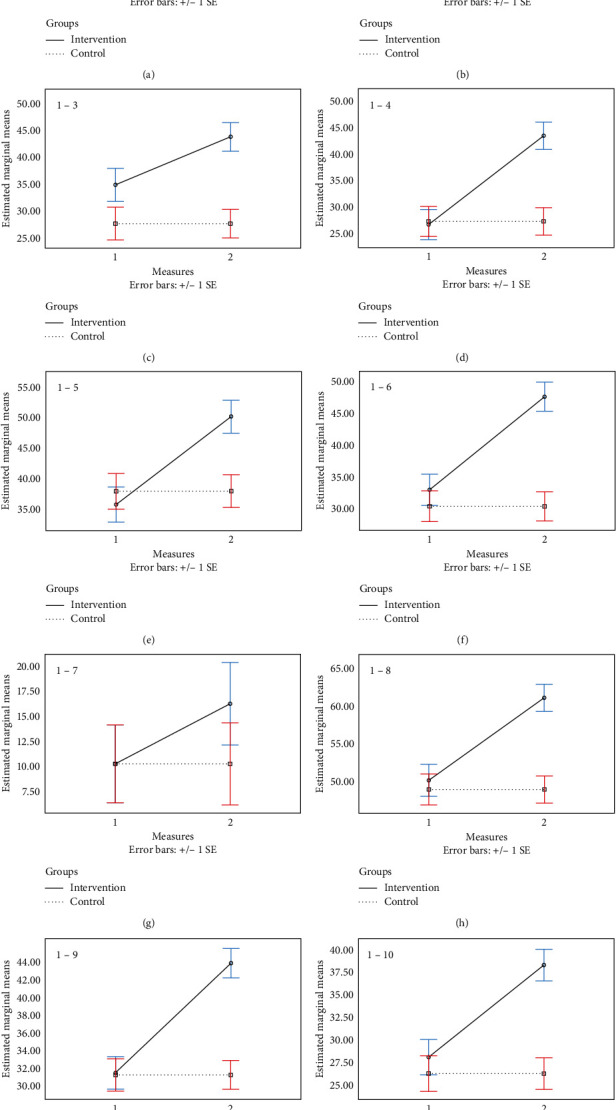
Interaction of time–group for measurements: (a) 1-1, physical function, (b) 1-2, role physical, (c) 1–3, body pain, (d) 1–4, general health, (e) 1–5, vitality, (f) 1–6, social functioning, (g) 1–7, role emotional, (h) 1–8, mental health, (i) 1–9, total physical health, and (j) 1–10, total mental health.

Based on the results of two-way RMANOVA, there were no significant time-group interaction (*P* > 0.05), main effect of time (*P* > 0.05), and intervention (*P* > 0.05). In addition, the results of ANCOVA showed that the changes in the physical function after intervention were not higher in the intervention group than that of in the control group (*P* > 0.05) as well as after adjusting for confounders (*P* > 0.05) (Table 2) (the chart is in the Figure2).

#### 2.8.4. Physical Role

Based on the results of two-way RMANOVA, there was a significant time-group interaction (*P* < 0.05). The results of the Sidak post hoc test indicated that, for the intervention group, there were significant differences between before and after intervention measurements (*P* < 0.05). Also, the results revealed a significant difference between intervention and control groups in after intervention measures (*P* < 0.05). In addition, the results of ANCOVA showed that the changes in the physical role after intervention were higher in the intervention group than that of in the control group (*P* < 0.05) as well as after adjusting for confounders (*P* < 0.05) (Table 2) (the chart is in the Figure 2).

#### 2.8.5. Body Pain

Based on the results of two-way RMANOVA, there was a significant time-group interaction (*P* < 0.05). The results of the Sidak post hoc test indicated that, for the intervention group, there were significant differences between before and after intervention measurements (*P* < 0.05). Also, the results revealed a significant difference between intervention and control groups in after intervention measures (*P* < 0.05). In addition, the results of ANCOVA showed that the changes in the body pain after intervention were higher in the intervention group than that of in the control group (*P* < 0.05) as well as after adjusting for confounders (*P* < 0.05) (Table 2) (The chart is in the Figure 2).

#### 2.8.6. General Health

Based on the results of two-way RMANOVA, there was a significant time-group interaction (*P* < 0.05). The results of the Sidak post hoc test indicated that, for the intervention group, there were significant differences between before and after intervention measurements (*P* < 0.05). Also, the results revealed a significant difference between intervention and control groups in after intervention measures (*P* < 0.05). In addition, the results of ANCOVA showed that the changes in the general health after intervention were higher in the intervention group than that of in the control group (*P* < 0.05) as well as after adjusting for confounders (*P* < 0.05) (Table 2) (the chart is in the Figure 2).

#### 2.8.7. Vitality

Based on the results of two-way RMANOVA, there was a significant time-group interaction (*P* < 0.05). The results of the Sidak post hoc test indicated that, for the intervention group, there were significant differences between before and after intervention measurements (*P* < 0.05). Also, the results revealed a significant difference between intervention and control groups in after intervention measures (*P* < 0.05). In addition, the results of ANCOVA showed that the changes in the vitality after intervention were higher in the intervention group than that of in the control group (*P* < 0.05) as well as after adjusting for confounders (*P* < 0.05) (Table 2) (the chart is in the Figure 2).

#### 2.8.8. Social Functioning

Based on the results of two-way RMANOVA, there was a significant time-group interaction (*P* < 0.05). The results of the Sidak post hoc test indicated that, for the intervention group, there were significant differences between before and after intervention measurements (*P* < 0.05). Also, the results revealed a significant difference between intervention and control groups in after intervention measures (*P* < 0.05). In addition, the results of ANCOVA showed that the changes in the social functioning after intervention were higher in the intervention group than that of in the control group (*P* < 0.05) as well as after adjusting for confounders (*P* < 0.05) (Table 2) (the chart is in the Figure 2).

#### 2.8.9. Emotional Role

Based on the results of two-way RMANOVA, there was a significant time-group interaction (*P* < 0.05). The results of the Sidak post hoc test indicated that, for the intervention group, there were significant differences between before and after intervention measurements (*P* < 0.05). Also, the results revealed a significant difference between intervention and control groups in after intervention measures (*P* < 0.05). In addition, the results of ANCOVA showed that the changes in the primary outcome in after intervention were higher in the intervention group than that of in the control group (*P* < 0.05) as well as after adjusting for confounders (*P* < 0.05) (Table 2) (the chart is in the Figure 2).

#### 2.8.10. Mental Health

Based on the results of two-way RMANOVA, there was a significant time-group interaction (*P* < 0.05). The results of the Sidak post hoc test indicated that, for the intervention group, there were significant differences between before and after intervention measurements (*P* < 0.05). Also, the results revealed a significant difference between intervention and control groups in after intervention measures (*P* < 0.05). In addition, the results of ANCOVA showed that the changes in the primary outcome after intervention were higher in the intervention group than that of in the control group (*P* < 0.05) as well as after adjusting for confounders (*P* < 0.05) (Table 2) (the chart is in the Figure 2).

#### 2.8.11. Total Physical Health

Based on the results of two-way RMANOVA, there was a significant time-group interaction (*P* < 0.05). The results of the Sidak post hoc test indicated that, for the intervention group, there were significant differences between before and after intervention measurements (*P* < 0.05). Also, the results revealed a significant difference between intervention and control groups in after intervention measures (*P* < 0.05). In addition, the results of ANCOVA showed that the changes in the primary outcome after intervention were higher in the intervention group than that of in the control group (*P* < 0.05) as well as after adjusting for confounders (*P* < 0.05) (Table 2) (the chart is in the Figure 2).

#### 2.8.12. Total Mental Health

Based on the results of two-way RMANOVA, there was a significant time-group interaction (*P* < 0.05). The results of the Sidak post hoc test indicated that, for the intervention group, there were significant differences between before and after intervention measurements (*P* < 0.05). Also, the results revealed a significant difference between intervention and control groups in after intervention measures (*P* < 0.05). In addition, the results of ANCOVA showed that the changes in the primary outcome after intervention were higher in the intervention group than that of in the control group (*P* < 0.05) as well as after adjusting for confounders (*P* < 0.05) (Table 2) (the chart is in the Figure 2).

#### 2.8.13. Total SF-36

Based on the results of two-way RMANOVA, there was a significant time-group interaction (*P* < 0.05). The results of the Sidak post hoc test indicated that, for the intervention group, there were significant differences between before and after intervention measurements (*P* < 0.05). Also, the results revealed a significant difference between intervention and control groups in after intervention measures (*P* < 0.05). In addition, the results of ANCOVA showed that the changes in the primary outcome after intervention were higher in the intervention group than that of in the control group (*P* < 0.05) as well as after adjusting for confounders (*P* < 0.05) (Table 2; Figure 1).

#### 2.8.14. Physician Visit

Based on the results of logistic regression after controlling the baseline measurements, there was a significant difference between intervention and control groups in after intervention measures (*P* < 0.05) as well as after adjusting for confounders (*P* < 0.05) (Table 3).

The results of Wilks' lambda multivariate tests for simultaneous comparing of subscales and scales showed that there were significant differences for QOL 8 subscales as well as QOL 2 scales between intervention and control groups (*P* < 0.05) (Table 4).

## 3. Discussion

This study investigates the effect of teach-back education on knowledge, performance, referral, and quality of life in heart failure patients. An important result of this study is knowledge and performance promotion in heart failure patients after teach-back education. This finding is significant immediately after education and 3 months after discharge, so that the effects of this education will last for up to 3 months after leaving the hospital. Consistent with this finding, in 44 heart failure patients referring to one of the hospitals in Ardabil province, Iran, Ghahramani et al. [22] show 4 sessions of self-care education, and each session for 20 minutes significantly increases knowledge and performance in heart failure patients [22]. Also, Mangolian [52] reports that four sessions of feedback self-care education combined with educational pamphlets would provide better knowledge and performance in heart failure patients up to one month after discharge [52]. Although these studies have evaluated the knowledge and performance of heart failure patients after self-care education at three or one month after discharge, however, these results are limited to the use of a pamphlet-based teaching approach and individual discussions with the patient. But in the present study, teach-back education was performed, and also, immediately after training and before discharge, a posttest was performed for more accurate monitoring of the effects of the intervention on the patients' knowledge and performance. In line with this finding, White et al. [33] showed the effects of teach-back education on the recalling of education in HF patients after one week of discharge [33]. The importance of the findings of our study is to track the patients' knowledge of heart failure after a longer period (i.e., one month); recalling information is less affected by recent patient memory. As the results show, changes in the mean scores of patients' knowledge significantly occur immediately after teach-back education, although it slightly increases after three months of discharge. Such a finding reflects the effects of teach-back education on an immediate basis, although this effect increased with a slow slope in the long term, so that three months after the education, the patients' knowledge increase almost twice as much as before the intervention (mean 10.03 vs. 5.23). This finding is confirmed after controlling for the baseline variables of age, a history of hypertension, smoking, and stress; the intervention and the control group have a significant difference in readmissions. In this regard, Howie-Esquivel et al. [53] state that after teach-back education, HF patients answered 75% of the teach-back questions correctly, and this amount of recall is related to the time spent in education during their hospital stay [53]. In our study, heart failure patients received three 30-minute education sessions; however, the present study does not investigate the impact of teach-back education sessions and its duration on the patients' knowledge and performance. Similarly, Sugathan et al. [54] showed that all HF patients trained by teach-back education recall and perform 90% of self-care measures and prioritize appropriate activities after discharge [54].

The third outcome variable in this study is the rate of readmissions of heart failure patients after teach-back education. Kaveh et al. [55] report that, during hospitalization, the patient's concern about recurrence of heart disease increases his/her adherence to self-care behaviors, but with the improvement of clinical conditions, it is predicted that recurrence and readmission will occur due to reduced adherence to treatment regimens [55]. Regarding readmission, the present study shows that teach-back education reduces the frequency of readmission of HF patients to the hospital. Consistent with this finding, a number of studies demonstrate the effectiveness of teach-back education in reducing hospital admissions in heart failure patients [8, 54, 56]. Also, there are studies with inconsistent results. In this regard, Ghahramani et al. [22] suggest that during the three months following self-care education, the frequency of readmissions of heart failure patients does not change [22] or Vidán et al. [57] argue that it is unclear whether self-care education can truly reduce readmission and may not even improve outcomes (quality of life, mortality, and readmission) in the elderly patients or those with depression disorder [57]. In any case, the authors of this study believe that the results of such studies are not a compelling reason for the ineffectiveness of teach-back education in heart failure patients; rather, given the results of improving patient self-care, the assumption that self-care is independent of the outcomes of HF patients, as the recent study suggests, may not seem reasonable.

Studies have shown the positive effects of self-care education on specific areas of quality of life, social interaction, mental functioning, self-efficacy, knowledge on symptoms of illness, diet, exercise, medications, and their side effects [58]. In this study, the teach-back method is considered as a new technique of self-care education. As previously mentioned, teach-back education is recommended as a strategy to confirm patients' understanding of health information and to promote health literacy [59]. In this regard, the results of the present study show the improvement of quality of life (except physical function) in patients with heart failure under self-care education through the teach-back method, so that after teach-back education, the quality of life in physical role, physical pain, general health, vitality, social function, emotional role, and mental health will be improved. One possible reason for the lack of improvement in physical function is the nature of the disease that restricts heart failure patients to perform their normal living activities. However, due to the fact that, in this study, the patients were included with ejection fraction less than 40% (i.e., severe heart failure), it is expected that the severity of the disease would reduce the effect of teach-back self-care education on their physical performance. As Izawa et al. [60] suggest different levels of disease severity in patients with heart failure affect health-related quality of life [60]. That is, patients' quality of life decreases with increasing severity of the disease in accordance with the NYHA classification and worsening of physiological symptoms. Therefore, it seems reasonable to expect that after three months of discharge due to the nature of the disease, its severity, and the age of the patients, physical performance is likely to be far from optimal. In general, improvement in most aspects of quality of life after teach-back education implies that this method of training is successful in heart failure patients. However, a careful multivariate analysis of variance shows simultaneously a significant difference in the means of the intervention and control groups in a combination of the dependent variable, the eight components of QOL. Thus, by controlling the pretest effect, the posttest scores for the relevant components of quality of life suggest improvement in the intervention and control patients. On the other hand, the results of binary regression analysis by controlling of baseline measurements such as age and stressgive more certainty about improving quality of life after teach-back education.

Findings indicate that patients experience a better quality of life because of their ability to perform self-care behaviors after teach-back education. This improvement of quality of life can be attributed to the increased knowledge and performance in HF patients. Apart from some of the drawbacks of the SF-36 in assessing quality of life in the elderly [61, 62], improving the quality of life in heart failure patients seems so real because the elderly patients with no cognitive impairment were selected, which would prevent the possible effects of this problem on the accuracy of quality of life measurement. Overall, the teach-back method as a patient-centered and a participatory educational approach fills the communication gap between nurses and patients, resulting in improved patient self-care and self-management [63]. In this regard, Borhani et al. [64] showed that quality of life of patients with heart failure is improved with participatory care [64]. Contrary to the findings of this study, Ross [45] measured quality of life of patients with heart failure after teach-back education using the Minnesota Heart Failure Questionnaire, and they concluded that patients' quality of life did not change significantly compared to preeducation because of the impact of learning on life and not heart failure itself [45].

Given the effectiveness of teach-back education on knowledge outcomes, performance, quality of life, and readmission, it is concluded that nurses can benefit from this method for effective education in heart failure patients. The authors of this article suggest that the effects of this training technique be compared with other predischarge training methods. It is also desirable for future research to evaluate the effect of teach-back education on the patients' attitude because patients' self-care behaviors, such as adherence to treatment regimens and quality of life, may be affected by changes in the patient's attitude to illness and life. Also, the researchers recommend that future studies should consider the possible reasons for not improving the physical function of patients after self-care education. Since most heart failure patients spend their recovery at home and with their families and given the importance of the family's role in helping to improve the patient's quality of life, the authors of this article suggest that in future studies, the family will also be present during the teach-back education and the self-care education provided in a group approach. It is also desirable to accurately monitor patient clinical outcomes, such as self-care behaviors, over specific periods of time through continuous telephone follow-ups.

One limitation of the study is that the patients were selected of heart failure cases based on the ejection fraction parameter (i.e., EF < 40%). Due to the fact that patients with different ejection fractions are similar in terms of several symptoms [65], the authors of this article recommend that future studies investigate the effects of the teach-back training method on a more comprehensive population of heart failure patients with different degrees of EF.

## Figures and Tables

**Table 1:** Study participants' characteristics in intervention and control groups.

Variables	Intervention (*n* = 35)		Control (*n* = 35)		*P* value
	*N* (mean)	% (SD)	*N* (mean)	% (SD)	
Gender, male (%)	24	68.6	24	68.6	1.000
Marital status, married (%)	34	97.1	35	100.0	1.000
Education, middle school (%)	18	51.4	14	40.0	0.523
Job, retired (%)	21	60.0	16	45.7	0.574
Living location, city (%)	23	65.7	23	65.7	1.000
Financial status, partially good (%)	19	54.3	15	42.9	0.212
Diabetic history, yes (%)	28	80.0	25	71.4	0.578
Hypertension history, yes (%)	29	82.9	28	80.0	1.000
Family history of cardiovascular disease, yes (%)	27	77.1	23	65.7	0.428
Hyperlipedimia history, yes (%)	19	54.3	22	62.9	0.628
Smoking history, no (%)	20	57.1	22	62.9	0.808
Alcoholic history, no (%)	33	94.3	34	97.1	1.000
Cardiac valve history, yes (%)	18	51.4	14	40.0	0.472
Hyper salt food, yes (%)	24	68.6	28	80.0	0.413
Hyper lipid food, yes (%)	18	51.4	22	62.9	0.469
Stressful personality, yes (%)	26	74.3	30	85.7	0.371
Dyspnea time, 24 pm–6 am (%)	14	40.0	9	25.7	0.075
Activity dyspnea, sleeping (%)	14	40.0	9	25.7	0.706
Dyspnea duration, less than 15 minutes (%)	18	51.4	19	54.3	0.811
Dyspnea number, between 4 and 5 times per 24 hours (%)	14	40.0	14	40.0	0.750
Referring sing, dyspnea (%)	14	40.0	18	51.4	0.130
Referring to physician after dyspnea, one day after occurrence (%)	15	42.9	12	34.3	0.629
Referring to physician, cardiologist (%)	24	68.6	25	71.4	0.386
Referred to center, public clinic (%)	16	45.7	16	45.7	0.120
How referred, with cooperation of family (%)	23	65.7	10	28.6	0.002

Age, year	66.7	18.9	65.6	7.7	0.754
Body mass index (BMI)	25.5	4.4	26.9	2.6	0.122
Systolic blood pressure (mmHg)	141.3	10.0	133.7	25.1	0.103
Diastolic blood pressure (mmHg)	95.9	10.6	92.8	12.0	0.267
Fasting blood sugar (FBS) (gr/dl)	169.6	55.3	156.1	53.3	0.300
Triglyceride	212.6	61.6	223.8	61.8	0.451
Cholesterol	189.7	53.0	201.1	47.8	0.345
Low-density lipoprotein	90.0	19.8	89.3	18.1	0.888
High-density lipoprotein	36.8	10.1	36.7	7.7	0.979
BUN	26.2	6.1	28.2	8.7	0.275
Creatinine	1.9	0.7	1.8	0.6	0.650

**Table 2:** Measurements of primary outcomes in intervention and control groups.

Variables		Intervention (*n* = 35)		Control (*n* = 35)		Mean difference	(95% CI) lower	(95% CI) lower	*P* value^#^	*P* value^##^	*P* value for interaction^###^
		Mean	SD	Mean	SD						
Knowledge	Before	5.23	2.67	4.37	2.16	0.86	−0.30	2.01	0.144	**<** **0.001**	**<** **0.001**
	After	9.37	2.34	5.43	1.77	3.31	2.78	3.86	**<** **0.001**		
	Post	10.03	2.04	8.00	1.64	1.63	0.94	2.32	**<** **0.001**		

Performance	Before	14.77	5.97	14.71	6.39	0.06	−2.89	3.01	0.969	**0.011**	**<** **0.001**
	After	25.26	5.50	16.74	5.68	8.47	7.42	9.51	**<** **0.001**		
	Post	28.14	4.91	26.06	3.36	2.06	0.82	3.29	**0.001**		

Physical function	Before	38.29	19.13	31.86	15.63	6.43	−1.90	14.76	0.128	**<** **0.001**	0.418
	After	39.29	16.77	31.86	15.63	1.60	−0.59	3.80	0.150		

Role physical	Before	5.71	17.24	7.86	17.96	−2.14	−10.54	6.25	0.612	**<** **0.001**	**<** **0.001**
	After	15.71	19.26	7.86	17.96	9.85	5.66	14.04	**<** **0.001**		

Body pain	Before	34.71	18.46	27.97	15.28	6.74	−1.34	14.83	0.101	**<** **0.001**	**0.001**
	After	43.09	14.13	27.97	15.28	10.07	6.30	13.84	**<** **0.001**		

General health	Before	26.94	15.80	27.51	16.38	−0.57	−8.25	7.10	0.882	**<** **0.001**	**<** **0.001**
	After	42.94	12.32	27.51	16.38	15.82	12.21	19.45	**<** **0.001**		

Vitality	Before	36.29	14.52	38.29	16.67	−2.00	−9.46	5.46	0.594	**<** **0.001**	**<** **0.001**
	After	49.43	11.99	38.29	16.67	12.74	9.94	15.56	**<** **0.001**		

Social functioning	Before	33.40	15.81	30.89	11.52	2.51	−4.09	9.11	0.450	**<** **0.001**	**<** **0.001**
	After	47.34	14.29	30.89	11.52	14.63	10.37	18.91	**<** **0.001**		

Role emotional	Before	10.40	20.95	10.43	22.50	−0.03	−10.40	10.34	0.996	**0.032**	**0.001**
	After	16.09	23.33	10.43	22.50	5.68	1.36	10.01	**0.011**		

Mental health	Before	50.63	10.58	49.49	11.92	1.14	−4.23	6.52	0.673	**<** **0.001**	**<** **0.001**
	After	60.69	7.10	49.49	11.92	10.37	8.54	12.20	**<** **0.001**		

Total physical health	Before	28.26	11.75	26.54	10.51	1.71	−3.60	7.03	0.522	**<** **0.001**	**<** **0.001**
	After	38.03	9.21	26.54	10.51	10.09	8.16	12.03	**<** **0.001**		

Total mental health	Before	31.60	10.78	31.37	9.38	0.23	−4.59	5.05	0.925	**<** **0.001**	**<** **0.001**
	After	43.26	8.80	31.37	9.38	11.70	9.61	13.79	**<** **0.001**		

Total SF-36	Before	29.51	10.99	28.00	8.59	1.51	−3.19	6.22	0.523	**<** **0.001**	**<** **0.001**
	After	39.31	8.77	28.00	8.59	10.06	8.31	11.81	**<** **0.001**		

^#^Independent *t*-test for baseline measures, ANCOVA for after (post) intervention measures. ^##^ANCOVA for after (post) intervention measures adjusted for gender, age, BMI, education, family history, hypertension history, diabetic history, hyperlipedimia history, smoking history, alcolic history, and stressful personality. ^###^Time-intervention interaction based on RMANOVA. Bold values indicate significant *P* values.

**Table 3:** Measurements of physician visit in intervention and control groups.

Variables		Frequency	Unadjusted						Adjusted^#^			
			Odds ratio	95% C.I.				*P* value	Odds ratio	95% C.I.		*P* value
				Lower			Upper			Lower	Upper	
Groups	Intervention	35	14.011	2.866		68.500		**<0.001**	45.456	5.623	367.473	**<0.001**
	Control	35	Referent	—		—		—	Referent	—	—	Referent

Physician visit												
>1 time in month		47	0.044	**0**.**008**			.242	**<0.001**	.011	.001	.144	**0.001**
One time in month		23	Referent	—			—	—	Referent	—	—	Referent

^#^Adjusted for baseline measurements, gender, age, BMI, education, family history, hypertension history, diabetic history, hyperlipedimia history, smoking history, alcoholic history, and stressful personality. Bold values indicate significant *P* values.

**Table 4:** Wilks' Lambda multivariate tests for simultaneous comparing of subscales and scales between intervention and control groups.

Effect	Wilks' lambda value	*F*	Hypothesis df	Error df	*P* value
QOL 8 subscales	0.368	13.109	8.000	61.000	**<0.001**
QOL 2 scales	0.442	42.279	2.000	67.000	**<0.001**

Bold values indicate significant *P* values.

## Data Availability

The data used to support the findings of this study are available from the first and corresponding authors upon request.
